# Biological significance and prognostic relevance of peripheral blood neutrophil-to-lymphocyte ratio in soft tissue sarcoma

**DOI:** 10.1038/s41598-018-30442-5

**Published:** 2018-08-10

**Authors:** Jason Yongsheng Chan, Zewen Zhang, Winston Chew, Grace Fangmin Tan, Chloe Liwen Lim, Lingyue Zhou, Wei Lin Goh, Eileen Poon, Nagavalli Somasundaram, Sathiyamoorthy Selvarajan, Kesavan Sittampalam, Francis Chin, Jonathan Teh, Mann Hong Tan, Khee Chee Soo, Melissa Teo, Mohamad Farid, Richard Quek

**Affiliations:** 1 Division of Medical Oncology, National Cancer Centre, Singapore, Singapore; 2 Cancer Science Institute of Singapore, National University of Singapore, Singapore, Singapore; 3 Department of Anatomical Pathology, Singapore General Hospital, Singapore, Singapore; 4 Division of Radiation Oncology, National Cancer Centre, Singapore, Singapore; 5 Division of Surgical Oncology, National Cancer Centre, Singapore, Singapore; 6 Duke-NUS Medical School, Singapore, Singapore

## Abstract

Peripheral blood indices of systemic inflammation such as the neutrophil-lymphocyte ratio (NLR) have been shown to be prognostic in various cancers. We aim to investigate the clinical significance of these indices in patients with soft tissue sarcoma (STS). Seven hundred and twelve patients with available blood counts at diagnosis and/or metastatic relapse were retrospectively examined. An optimal cutoff for NLR-high (>2.5) in predicting overall survival (OS) was determined using receiver operating curve analyses. Survival analyses were performed using the Kaplan-Meier method and multivariate Cox proportional models. Our results show that NLR was significantly higher in patients with distant metastasis at diagnosis (n = 183) compared to those without (n = 529) (median: 4.36 vs 2.85, *p* < 0.0001). Progression of localized disease at diagnosis to metastatic relapse within the same patients was associated with an interval increase in NLR (median: 3.21 vs 3.74, *p* = 0.0003). In multivariate analysis, NLR-high was the only consistent factor independently associated with both worse OS (HR 1.53, 95% CI 1.10–2.13, *p* = 0.0112) and relapse-free survival (HR 1.41, 95% CI 1.08–1.85, *p* = 0.0125) in localized disease, as well as OS (HR 1.82, 95% CI 1.16–2.85, *p* = 0.0087) in metastatic/unresectable disease. In conclusion, high NLR is an independent marker of poor prognosis among patients with STS.

## Introduction

Soft tissue sarcomas represent a heterogeneous group of tumors originating from mesenchymal precursors that exhibit histopathological diversity and varying levels of biological aggressiveness^1^. In patients with localized disease, up to 50% eventually experience metastases and death despite undergoing definitive therapy^2^. Notwithstanding advances in modern chemotherapy, prognosis remains dismal in metastatic disease, with a two-year overall survival (OS) rate of approximately 30%. Therefore, there is currently an unmet need for a greater understanding of factors which determine the biological behavior and prognosis of soft tissue sarcomas, on top of already established parameters such as anatomical location, tumor size, tumor depth, pathologic grade, histologic subtype, and margin status^3,4^.

The inflammatory response is a hallmark of cancer and is known to affect every single step of tumorigenesis from tumor initiation and promotion, to metastatic progression^5,6^. In addition, cancer-related inflammation contributes to subversion of host immune surveillance, induction of genetic instability, and reduced therapeutic response^7^. Recently, emerging evidence supports an important role of systemic inflammation in the pathobiology of soft tissue sarcomas, with several studies reporting prognostic implications of inflammatory biomarkers such as serum cytokines^8^, C-reactive protein (CRP)^9^, erythrocyte sedimentation rate (ESR)^10^, as well as alterations in specific subsets of circulating peripheral blood cells. The potential clinical utility of elevated neutrophil-lymphocyte ratio (NLR)^11–14^, decreased lymphocyte-monocyte ratio (LMR)^15^ or raised platelet-lymphocyte ratio (PLR)^16^ as adverse prognostic indicators in localized soft tissue sarcomas has been suggested in several studies, albeit with mixed results. Amongst these indices, the NLR has been the most thoroughly investigated, and recent meta-analyses have shown that high NLR is correlated with poor survival across several cancer types besides sarcomas^17,18^. The correlation of these indices with biological characteristics, including the temporal relationship with metastatic progression, as well as the prognostic significance in metastatic disease however, remain unclear.

Therefore, we conducted a retrospective study to investigate the prognostic relevance and clinical correlates of peripheral blood indices of systemic inflammation, including the NLR, PLR, and LMR, across various stages and histological subtypes of soft tissue sarcoma.

## Results

### Patient demographics

The median age at diagnosis was 56 years (range: 14 to 95 years). Three hundred and forty-six (48.6%) were male and 366 (51.4%) were female. Out of all patients, 54.4% (*n* = 387) had high-grade tumors, 13.5% (*n* = 96) had low-grade tumors, 23.3% (*n* = 166) had intermediate-grade tumors, while the rest were unknown. The histopathological subtypes included undifferentiated pleomorphic sarcoma (n = 151), liposarcoma (n = 150), leiomyosarcoma (n = 111), angiosarcoma (n = 76), synovial sarcoma (n = 50), myxofibrosarcoma (n = 43) and others (n = 131). Out of 529 (74.3%) patients with localized disease, 473 patients underwent surgical resection with curative intent. The median tumor size was 9.4 cm (range: 0.9 to 55 cm). Tumors were resected with R0 margins in 295 cases (62.4%) and with R1 margins in 166 cases (35.1%). Post-operative radiation therapy was administered to 178 patients (37.6%). Post-operative chemotherapy was administered to 30 patients (6.3%). Ten patients (2.1%) received neoadjuvant chemotherapy and 6 (1.3%) received neoadjuvant radiotherapy. Patient characteristics are summarized in Table 1.

**Table 1:** Clinicopathological features and neutrophil-lymphocyte ratio at diagnosis.

Characteristic (n)	Neutrophil-lymphocyte ratio at diagnosis (%)		*p*
	≤2.5	>2.5	
Total (712)	254 (35.7%)	458 (64.3%)	—
*Sex*			
Male (346)	124 (35.8%)	222 (64.2%)	0.929
Female (366)	130 (35.5%)	236 (64.5%)	
*Age at diagnosis (years)*			
>65 (212)	67 (31.6%)	145 (68.4%)	0.14
≤65 (500)	187 (37.4%)	313 (62.6%)	
*Cardiovascular co-morbidities* ^†^			
Present (256)	94 (36.7%)	162 (63.3%)	0.663
Absent (456)	160 (35.1%)	296 (64.9%)	
*Ethnicity*			
Chinese (558)	199 (35.7%)	359 (64.3%)	0.991
Other (154)	55 (35.7%)	99 (64.3%)	
*Histology*			
Undifferentiated pleomorphic sarcoma (151)	42 (27.8%)	109 (72.2%)	0.253
Liposarcoma (150)	67 (44.7%)	83 (55.3%)	
Leiomyosarcoma (111)	32 (28.8%)	79 (71.2%)	
Angiosarcoma (76)	25 (32.9%)	51 (67.1%)	
Synovial sarcoma (50)	20 (40.0%)	30 (60.0%)	
Myxofibrosarcoma (43)	20 (46.5%)	23 (53.5%)	
Miscellaneous* (131)	48 (36.6%)	83 (63.4%)	
*Distant metastasis at diagnosis*			
Present (183)	37 (20.2%)	146 (79.8%)	<0.0001
Absent (529)	217 (41.0%)	312 (59.0%)	
*Tumor grade*			
G3 (387)	108 (27.9%)	279 (72.1%)	<0.0001
G2 (166)	67 (40.4%)	99 (59.6%)	
G1 (96)	56 (58.3%)	40 (41.7%)	
*Tumor size^*			
>5 cm (389)	148 (38.0%)	241 (62.0%)	0.0035
≤5 cm (127)	67 (52.8%)	60 (47.2%)	
*Tumor depth^*			
Deep (405)	152 (37.5%)	253 (62.5%)	0.003
Superficial (111)	59 (53.2%)	52 (46.8%)	
*Platelet-lymphocyte ratio*			
>182 (364)	41 (11.3%)	323 (88.7%)	<0.0001
≤182 (348)	213 (61.2%)	135 (38.8%)	
*Lymphocyte-monocyte ratio*			
≤2.4 (253)	10 (4.0%)	243 (96.0%)	<0.0001
>2.4 (459)	244 (53.2%)	215 (46.8%)	

^†^Includes hypertension, hyperlipidemia, diabetes mellitus, ischemic heart disease, cerebrovascular disease.

*Miscellaneous sarcomas include solitary fibrous tumor (n = 26), malignant peripheral nerve sheath tumor (n = 16), endometrial stromal sarcoma (n = 14), epithelioid sarcoma (n = 12), intimal sarcoma (n = 9), undifferentiated endometrial sarcoma (n = 8), fibromyxoid sarcoma (n = 7), alveolar soft part sarcoma (n = 6), fibrosarcoma (n = 6), clear cell sarcoma (n = 5), myofibrosarcoma (n = 4), extraskeletal chondrosarcoma (n = 4), myofibroblastic sarcoma (n = 4), epithelioid hemangioendothelioma (n = 2), PEComa (n = 2), low grade spindle cell tumor (n = 2), desmoplastic small round cell tumor (n = 1), embryonal sarcoma (n = 1), fibromyxoid tumor (n = 1), malignant round cell tumor (n = 1).

^Non-metastatic cases only.

Across the entire cohort, the values for NLR (median: 3.14, range: 0.82 to 68.57), PLR (median: 184.5, range: 6 to 10258) and LMR (median: 2.98, range: 0.02 to 18.46) follow non-normal distributions (all *p* < 0.0001). Patients were dichotomized according to levels of NLR, PLR, LMR using optimized cut-offs to predict OS as derived from ROC curve analysis (>2.5, >182 and ≤2.4, respectively). The areas under the curve for NLR, PLR and LMR for OS were 0.660 (95% CI 0.624 to 0.695), 0.604 (95% CI 0.567 to 0.640), and 0.652 (95% CI 0.616 to 0.687), respectively. Four hundred and fifty-eight patients (64.3%) were categorized as NLR-high, 364 (51.1%) as PLR-high, and 459 (64.5%) as LMR-high.

### Clinicopathological correlates

**Figure 1:**
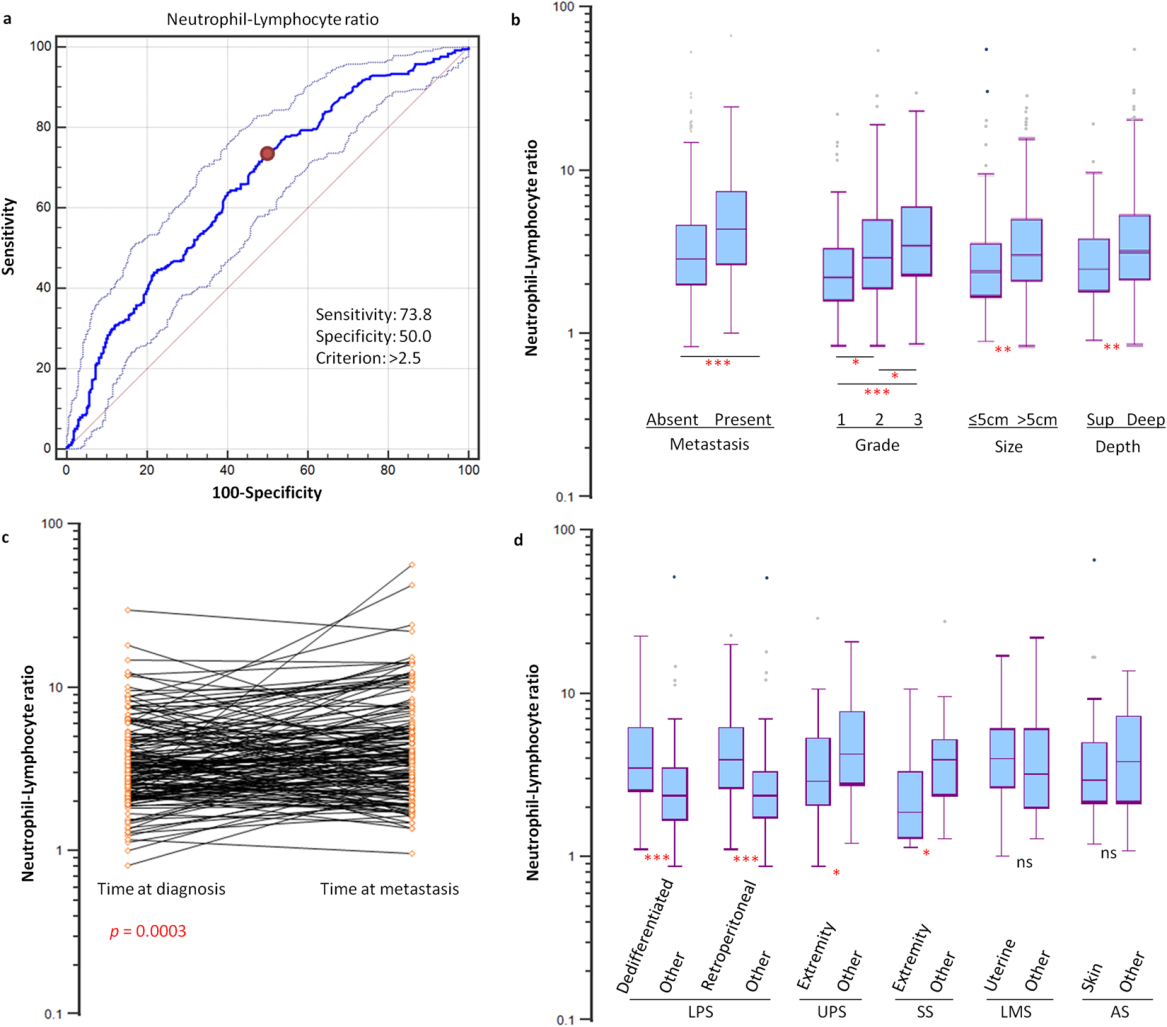
Derivation of NLR and correlation with clinicopathology. (**a**) An optimal cut-off for high NLR (>2.5) in predicting overall survival was determined using receiver operating curve analyses. (**b**) Patients with distant metastasis at diagnosis, higher tumor grade, larger tumor size, as well as tumor depth were associated with higher NLR. (**c**) Progression of localized disease at diagnosis to metastatic relapse within the same patients was associated with an increase in NLR (*p* = 0.0003, Wilcoxon signed-rank test). (**d**) In histotype-specific analysis, liposarcomas (LPS) with dedifferentiated components or retroperitoneal primary sites were significantly associated with higher NLR. Undifferentiated pleomorphic sarcomas (UPS) and synovial sarcomas (SS) of the extremities had lower NLR. No difference was observed for uterine leiomyosarcomas (LMS) or skin angiosarcomas (AS) compared to other primary sites. *p < 0.05, **p < 0.001, ***p < 0.0001 by Mann-Whitney U test. ns, non-significant; sup, superficial.

NLR-high was significantly associated with distant metastasis at diagnosis (*p* < 0.0001), high tumor grade (*p* < 0.0001), tumor size >5 cm (*p* = 0.0035), deep tumors (*p* = 0.0030), PLR-high (*p* < 0.0001) and LMR-low (*p* < 0.0001), but not with sex, age at diagnosis, cardiovascular co-morbidities or ethnicity (Table 1). NLR was significantly higher in patients with distant metastatic disease at diagnosis compared to those without (median: 4.36 vs 2.85, *p* < 0.0001) (Fig. 1b). This was accompanied by both higher levels of neutrophils (median: 6.74 vs 4.68, *p* < 0.0001) as well as lower levels of lymphocytes (median: 1.51 vs 1.66, *p* = 0.0789). Amongst patients with non-metastatic disease at the time of diagnosis, 167 (35.3%) eventually developed distant metastasis after a median of 11.2 months, out of which 155 had available pre-treatment peripheral blood neutrophil and lymphocyte counts at the time of diagnosis and at the time of metastatic relapse. Progression of localized disease at diagnosis to metastatic relapse in these patients was associated with an interval increase in NLR (median: 3.21 vs 3.74, *p* = 0.0003) (Fig. 1c). This observation was mainly related to an interval reduction in lymphocyte counts (median: 1.61 vs 1.41, *p* = 0.0001) rather than an increase in neutrophil counts (median: 4.96 vs 5.27, *p* = 0.529).

In histotype-specific analysis, liposarcomas with dedifferentiated components or those derived from retroperitoneal sites were significantly associated with higher NLR (dedifferentiated vs other: 3.49 vs 2.37, *p* < 0.0001; retroperitoneal vs other: 3.93 vs 2.35, *p* < 0.0001). Undifferentiated pleomorphic sarcomas (UPS) and synovial sarcomas (SS) of the extremities were associated with lower NLR, in comparison to those arising from non-extremity sites (UPS: 2.81 vs 4.31, *p* = 0.0073; SS: 1.90 vs 3.92, *p* = 0.0081). No difference was observed for leiomyosarcomas of the uterus or angiosarcomas of the skin compared to those arising from other primary sites (Fig. 1d).

### Survival analyses

**Figure 2:**
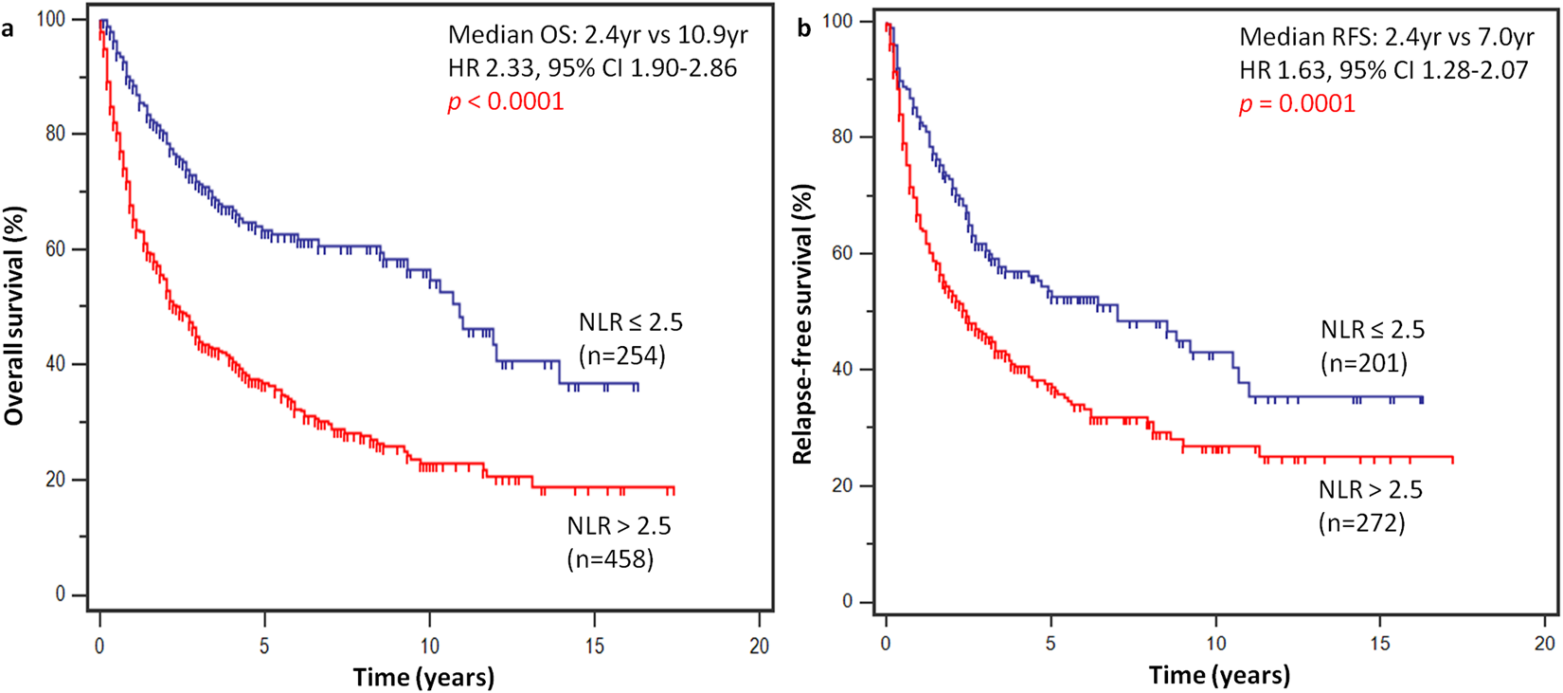
Survival outcomes stratified by NLR. High NLR was associated with worse (**a**) overall survival and (**b**) relapse-free survival.

At the time of data analysis, 378 patients (53.1%) had died. In the overall cohort, NLR-high at baseline prior to any therapy (including surgery) was associated with worse OS (HR 2.33, 95% CI 1.90 to 2.86, *p* < 0.0001) (Fig. 2). Median OS was 2.4 years in patients with NLR-high and 10.9 years with NLR-low. In the subgroup of patients with localized resectable disease who underwent curative surgery, NLR-high was correlated with worse OS (HR 1.93, 95% CI 1.45 to 2.57, *p* < 0.0001) and RFS (HR 1.63, 95% CI 1.28 to 2.07, *p* < 0.0001), as were advanced age at diagnosis, high tumor grade, large tumor size, positive surgical margins, high PLR and low LMR. Similarly, subgroup analysis of patients with metastatic/unresectable disease showed that NLR-high had a detrimental effect on OS (HR 2.19, 95% CI 1.62 to 2.97, *p* < 0.0001) (Table 2). In multivariate models adjusted for clinicopathological predictors of survival, NLR-high was the only consistent factor independently associated with both worse OS (HR 1.53, 95% CI 1.10 to 2.13, *p* = 0.0112) and RFS (HR 1.41, 95% CI 1.08 to 1.85, *p* = 0.0125) in localized disease, as well as OS (HR 1.82, 95% CI 1.16 to 2.85, *p* = 0.0087) in metastatic/unresectable disease. In metastatic/unresectable cases, LMR-low was also independently associated with worse OS (HR 1.71, 95% CI 1.21 to 2.43, *p* = 0.0026). Other independent predictors of survival outcomes are summarized in Table 3.

**Table 2:** Univariate survival analysis.

Characteristic	Localized				Metastatic/Unresectable	
	Relapse-free survival		Overall survival		Overall survival	
	HR (95% CI)	*p*	HR (95% CI)	*p*	HR (95% CI)	*p*
Sex (male *vs* female)	1.22 (0.96 to 1.56)	0.105	1.09 (0.82 to 1.46)	0.530	1.26 (0.95 to 1.68)	0.0918
Age at diagnosis (>65 *vs* ≤65 years)	1.32 (0.99 to 1.74)	0.0387	1.70 (1.21 to 2.39)	0.0005	1.39 (1.03 to 1.89)	0.0175
Ethnicity (Chinese *vs* other)	0.75 (0.55 to 1.04)	0.0541	0.85 (0.58 to 1.24)	0.372	0.92 (0.62 to 1.38)	0.669
Cardiovascular co-morbidities (Present *vs* absent)	1.24 (0.96 to 1.61)	0.0841	1.41 (1.04 to 1.91)	0.0179	1.17 (0.87 to 1.57)	0.263
Tumor grade (3 *vs* 1–2)	2.90 (2.27 to 3.70)	<0.0001	3.68 (2.76 to 4.91)	<0.0001	1.25 (0.91 to 1.73)	0.172
Tumor size (>5 cm *vs* ≤5 cm)	1.76 (1.34 to 2.30)	0.0003	1.58 (1.14 to 2.18)	0.0126	—	—
Tumor depth (deep *vs* superficial)	1.16 (0.86 to 1.57)	0.355	0.94 (0.65 to 1.36)	0.729	—	—
Surgical margins (R1 *vs* R0)	1.47 (1.13 to 1.91)	0.0020	1.42 (1.04 to 1.93)	0.0180	—	—
NLR at diagnosis (>2.5 *vs* ≤2.5)	1.63 (1.28 to 2.07)	0.0001	1.93 (1.45 to 2.57)	<0.0001	2.19 (1.62 to 2.97)	<0.0001
PLR at diagnosis (>182 *vs ≤*182)	1.65 (1.29 to 2.10)	<0.0001	1.70 (1.28 to 2.26)	0.0003	1.70 (1.28 to 2.26)	0.0001
LMR at diagnosis (≤2.4 *vs* >2.4)	1.71 (1.28 to 2.29)	<0.0001	1.85 (1.31 to 2.59)	<0.0001	2.04 (1.53 to 2.73)	<0.0001
Chemotherapy* (Yes *vs* No)	1.08 (0.69 to 1.70)	0.718	1.10 (0.65 to 1.87)	0.714	0.67 (0.49 to 0.92)	0.0067
Radiotherapy* (Yes *vs* No)	0.84 (0.66 to 1.07)	0.150	0.99 (0.74 to 1.32)	0.956	0.91 (0.68 to 1.21)	0.485

*Neoadjuvant or adjuvant for localized disease, palliative for metastatic/unresectable disease.

**Table 3:** Multivariate survival analysis.

Characteristic	Localized				Metastatic/Unresectable	
	Relapse-free survival		Overall survival		Overall survival	
	HR (95% CI)	*p*	HR (95% CI)	*p*	HR (95% CI)	*p*
Age at diagnosis (>65 *vs* ≤65 years)	—	—	1.64 (1.19 to 2.26)	0.0026	1.40 (1.02 to 1.92)	0.0379
Tumor grade (3 *vs* 1–2)	2.78 (2.08 to 3.70)	<0.0001	3.38 (2.36 to 4.85)	<0.0001	—	—
Tumor size (>5 cm *vs ≤*5 cm)	1.56 (1.13 to 2.15)	0.0070	1.48 (1.01 to 2.16)	0.0419	—	—
Surgical margins (R1 *vs* R0)	1.36 (1.05 to 1.75)	0.0200	—	—	—	—
NLR at diagnosis (>2.5 *vs ≤*2.5)	1.41 (1.08 to 1.85)	0.0125	1.53 (1.10 to 2.13)	0.0112	1.82 (1.16 to 2.85)	0.0087
LMR at diagnosis (≤2.4 *vs* >2.4)	—	—	—	—	1.71 (1.21 to 2.43)	0.0026
Palliative chemotherapy (Yes *vs* No)	—	—	—	—	0.62 (0.46 to 0.85)	0.0027

**Figure 3:**
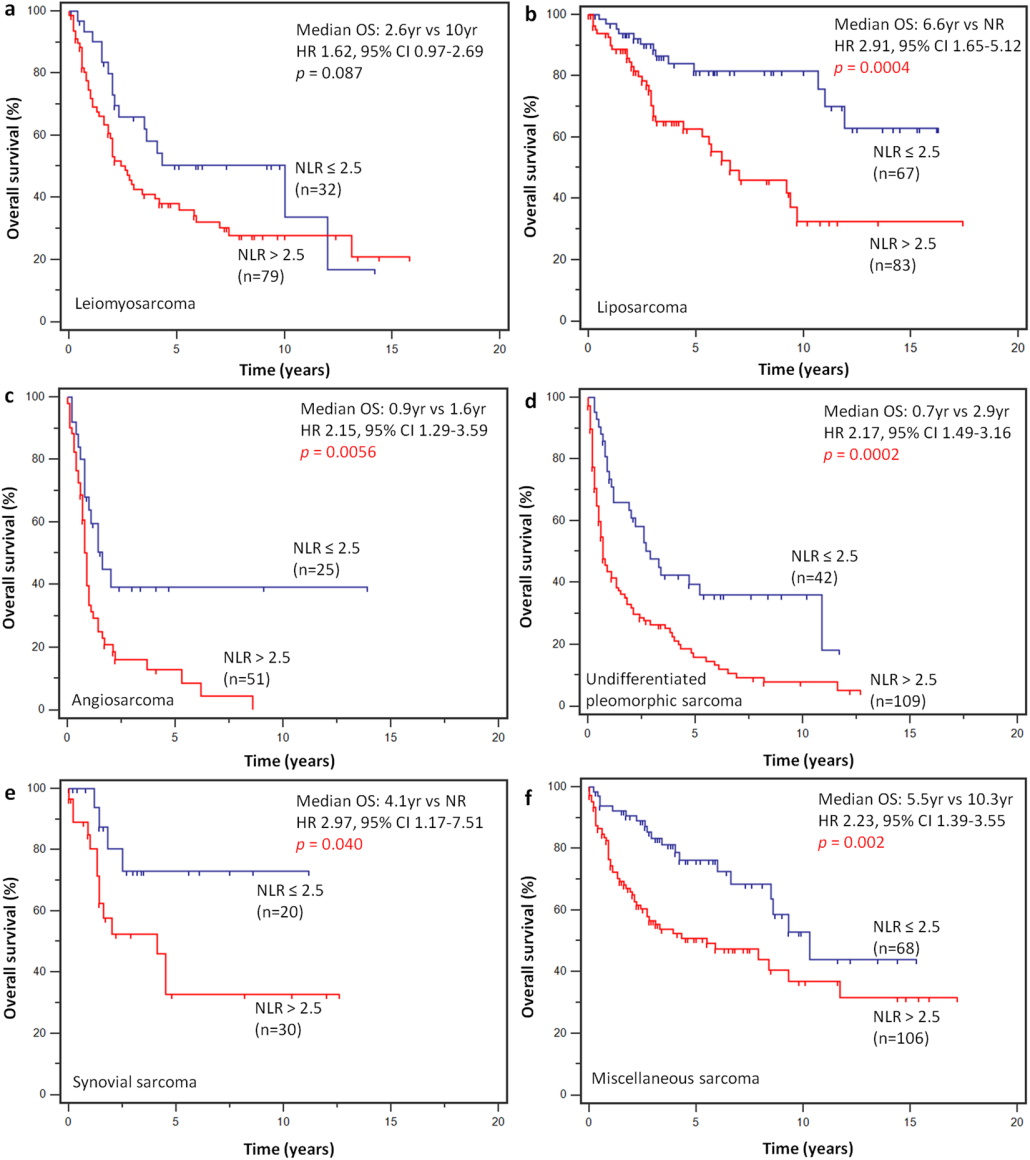
Survival analysis by histological subtypes. High NLR was associated with worse overall survival across sarcoma histotypes, including (**a**) leiomyosarcoma (n = 111), (**b**) liposarcoma (n = 150), (**c**) angiosarcoma (n = 76), (**d**) undifferentiated pleomorphic sarcoma (n = 151), (**e**) synovial sarcoma (n = 50) and (**f**) other miscellaneous sarcomas (n = 174).

**Figure 4:**
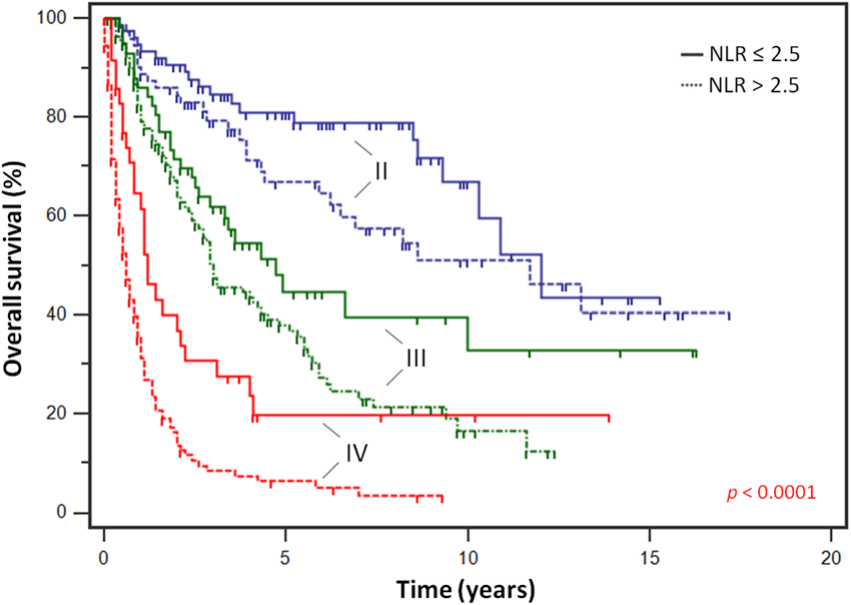
Survival outcomes by stage. Analysis of overall survival according to American Joint Committee on Cancer (AJCC) stages subdivided by NLR revealed a significant worse prognosis for NLR-high subgroups, with a 1.6 fold, 1.5 fold and 2.0 fold risk of death within stages II (HR 1.55, 95% CI 1.10–2.19), III (HR 1.55, 95% CI 1.01–2.37), and IV (HR 2.03, 95% CI 1.14–3.62), respectively.

In subgroup analysis according to histotype, NLR-high was associated with worse OS across different sarcoma subtypes (Fig. 3). Among patients with localized disease treated with curative surgery, a total of 441 patients (93.2%) had complete data on tumor stage - stage I (n = 88), stage II (n = 152) and stage III (n = 201). Analysis of OS according to AJCC stages subdivided by NLR revealed a significantly worse prognosis for NLR-high subgroups, with a 1.6 fold, 1.5 fold and 2.0 fold risk of death in patients who underwent curative surgery with stages II (HR 1.55, 95% CI 1.10 to 2.19) and III (HR 1.55, 95% CI 1.01 to 2.37) disease, as well as within stage IV (HR 2.03, 95% CI 1.14 to 3.62), respectively (log-rank *p* < 0.0001) (Fig. 4).

## Discussion

Our current study demonstrates that peripheral blood indices of systemic inflammation are potential biomarkers of poor prognosis in soft tissue sarcoma. Specifically, we showed that a high NLR independently correlated with poor survival outcomes in patients across both localized and metastatic stages of disease. This association was consistently observed across different histological subtypes, suggesting that systemic inflammation, as represented by an elevated NLR, is a fundamental adverse phenomenon in soft tissue sarcoma. Notably, we observed that NLR was significantly higher in patients with tumors of higher grade, depth, and larger size. In addition, we showed that NLR was more pronounced in the presence of distant metastasis - in both *de novo* and relapsed settings.

In line with our observations, previous studies have shown that high NLR in the pre-operative setting was associated with worse survival outcomes in non-metastatic soft tissue sarcomas^11–14^. In a small study of 83 patients with miscellaneous surgically-excised soft tissue sarcomas by Idowu *et al*., high NLR ≥ 5 was an independently associated with poor OS^11^. Nakamura *et al*. examined the role of pre-treatment CRP levels and NLR in a cohort of 129 patients with non-metastatic disease, and observed significantly worse disease-specific survival outcomes for patients with both an elevated CRP level and high NLR, as compared to those with both a normal CRP level and low NLR^12^. These results were subsequently corroborated in larger studies, which similarly showed significant associations with faster time to tumor recurrence and poorer OS^13^, as well as disease-specific survival^14^ [Table 4]. Other investigators incorporating related inflammatory indices into their studies, in addition to NLR - including the PLR, LMR or ESR likewise showed that these were prognostic in localized soft tissue sarcomas^10,15,16^. The prognostic role of PLR has been investigated in a meta-analysis which concluded that high PLR was independently associated with worse OS in various solid tumors^17^, as well as in a retrospective analysis of resected soft tissue sarcoma^16^. Pre-operative LMR, but not NLR or PLR, has also been independently correlated with worse disease-free survival and disease-specific survival in one study on resected soft tissue sarcoma^15^. Our own results showed that high NLR, but not PLR or LMR, was independently associated with worse RFS and OS. In addition, both NLR and LMR were independent predictors for worse OS in metastatic/unresectable soft tissue sarcoma. Taken together, it appears that the NLR may be a reliable prognostic biomarker in soft tissue sarcoma.

**Table 4:** Overview of soft tissue sarcoma studies on the impact of survival outcomes by a high NLR.

Reference	n	Measurements	NLR Cut-off^†^	Associations with survival outcomes in multivariate analysis
^11^	83	Pre-operative NLR	5	High NLR with worse OS
^12^	129	Pre-treatment NLR, CRP	2.3*	High NLR, CRP with worse DSS
^13^	260	Pre-operative NLR	3.45 for TTR 3.58 for OS	High NLR with worse TTR and OS
^14^	818	Pre-operative NLR, CRP, hemoglobin, albumin	5.3**	High NLR with worse DSS
^10^	162	Pre-operative NLR, CRP, ESR	2.5	High ESR, CRP with worse DSS
^15^	340	Pre-operative NLR, PLR, LMR	5***	High LMR with worse DFS and DSS
^16^	222	Pre-operative NLR, PLR	2.5	High PLR with worse DFS and OS

Abbreviations: NLR, neutrophil-lymphocyte ratio; CRP, C-reactive protein; ESR, erythrocyte sedimentation rate; PLR, platelet-lymphocyte ratio; LMR, lymphocyte-monocyte ratio; OS, overall survival; DSS, disease-specific survival; TTR, time to recurrence; DFS, disease-free survival.

^†^Selected by ROC analysis unless otherwise stated.

*Based on median value in their dataset.

**Based on institution reference values.

***Based on published data.

To date, it remains unclear if alterations in the proportion of neutrophil and lymphocyte counts directly influence disease biology, or simply represent a non-specific inflammatory reaction to malignant progression. Regardless, our results suggest that NLR is a dynamic measure of systemic inflammation that changes longitudinally with the course of disease - patients who developed metastatic relapse demonstrated an interval increase in NLR as compared to the time when they were initially diagnosed with localized disease, supporting preliminary observations by other investigators^10^. Such temporal changes have also been reported in the setting of metastatic soft tissue sarcoma, in which an interval increase in NLR after initial treatment was associated with worse progression-free survival and OS^19^. It is well known that tumor-infiltrating lymphocytes may limit the metastatic cascade of cancer cells^20^, while neutrophils may contribute to tumor cell migration and metastasis by remodeling the extracellular matrix^21^ and promotion of angiogenesis^20^. Furthermore, neutrophils may facilitate tumor immune escape by inhibition of cytotoxic T lymphocytes^22^ and lymphokine-activated killer cells^23^. Given these opposing roles of neutrophils and lymphocytes in the immune regulation of tumor cells, it is tempting to speculate that an elevated NLR provides for an optimal immune milieu to promote tumor dissemination and metastasis.

Our present study is limited by its retrospective design and patient cohort derived from a single institution. Data on individual co-morbidities may not be completely captured and other inflammatory markers known to be prognostic in soft tissue sarcoma, such as CRP, ESR or albumin were also unavailable. It has been suggested that the NLR may be affected by medical conditions including hypertension, diabetes mellitus, hyperlipidemia, coronary artery disease, chronic kidney disease, heart failure, thyroid dysfunction, cerebrovascular disease and peripheral arterial disease^24^. Nonetheless, our study represents one of the largest to date and includes patients across all stages of disease. External validation on other retrospective datasets and prospective studies on independent group of patients would be necessary to verify our findings.

In conclusion, our study suggests that peripheral blood indices of systemic inflammation, as measured by the NLR, may be a useful prognostic factor in patients with soft tissue sarcoma.

## Patients and Methods

### Study cohort

Medical records of all patients with histologically-proven soft tissue sarcoma consecutively seen at the National Cancer Centre Singapore between April 1998 and June 2016 were retrospectively reviewed. A total of 712 patients who had available pre-treatment (prior to any therapy including surgery) peripheral blood neutrophil and lymphocyte counts at the time of diagnosis and/or metastatic relapse were included in the final analysis. None of the included patients had evidence of an infectious process or a hematological disorder at the time of blood draw. Median follow-up was 25.8 months. Clinicopathological information available included sex, age, ethnicity, tumor location, presence of distant metastasis, tumor size, tumor depth, histological subtype, tumor grade and surgical margins. Sex, age and ethnicity of the subjects were verified against their National Registry Identification Cards. Tumor size was defined as either the largest diameter as measured in resected pathological specimens or as measured from cross-sectional radiographs. Histological grading was performed using the *Fédération Nationale des Centres de Lutte Contre le Cancer* (FNCLCC) grading system^25^. Positive (R1) or negative (R0) surgical margins were defined depending upon microscopic involvement on histopathological analysis. All histological parameters were characterized by two independent expert soft tissue sarcoma pathologists (S.S., K.S.). Staging was based on the American Joint Committee on Cancer (AJCC) 7^th^ edition classification^26^. These data were obtained at the time of diagnosis and at subsequent follow-up. Patient characteristics are summarized in Table 1. This work was done under approval from the SingHealth Centralized Institution Review Board. Informed consent was obtained from all participants and/or their legal guardians. All methods were performed in accordance with the relevant guidelines and regulations. The datasets generated during and/or analysed during the current study are available from the corresponding author on reasonable request.

### Statistical analysis

The NLR was calculated by dividing absolute neutrophil counts by absolute lymphocyte counts taken at the same sitting. Receiver operating characteristic (ROC) curve analysis via the method of DeLong *et al*.^27^ was used to identify the optimal discriminatory cut-off value for NLR as a univariable predictor of OS. In previous studies, empirical cut-offs for NLR had been derived using ROC curve analyses^10,11,13,16^ while others had arbitrarily based their cut-off on median values^12^, institution references^14^, or previously published data^15^. These studies had not demonstrated any consistent or validated cut-off values, and were highly variable (ranging from 2.3 to 5.3) (summarized in Table 4). Given the heterogeneous methodologies and variable cut-offs obtained in these studies, we thus selected the ROC curve analysis as the most objective statistical method for our study. Internal validation was done using the bootstrapping method with 1000 iterations. Cutoffs for PLR and LMR were similarly obtained. The Kolmogorov–Smirnov test was applied to assess the normality of distribution for NLR, PLR and LMR. Comparisons of the frequencies of categorical variables were performed using Pearson’s Chi-squared tests. Continuous variables were represented by Box-Whisker plots and their associations with NLR levels were evaluated by Mann-Whitney U tests. The Wilcoxon sign-ranked test was used for pair-wise comparisons. The primary and secondary survival endpoints of interest in this study are OS and relapse-free survival (RFS), respectively. For analyses of OS, survival was measured from the date of diagnosis till the date of death from any cause, or was censored at the date of the last follow-up for survivors. RFS was defined as the time elapsed from the date of definitive surgery till the date of relapse or death from any cause. Kaplan-Meier survival curves were plotted to estimate actuarial survival, and compared using the log-rank test. Cox proportional hazards regression was used to calculate hazard ratios (HR) with corresponding 95% confidence intervals (95% CI) of mortality according to various clinicopathological features. Multivariate Cox regression model via a stepwise procedure was used to test for independence of significant factors identified on univariate analysis. All statistical evaluations were made assuming a two-sided test with significance level of 0.05 unless otherwise stated. All tests were performed using MedCalc statistical software for Windows version 17.9 (MedCalc Software, Ostend, Belgium).
